# An Improved Ferrozine-Based Protocol for Safe, Reproducible, and Accurate Quantification of Iron in Biological and Chemical Samples

**DOI:** 10.3390/mps9010009

**Published:** 2026-01-09

**Authors:** Chao Wang, Shan Zhang

**Affiliations:** 1 School of Materials Science and Engineering, Institute of Materials Science and Devices, Suzhou University of Science and Technology, Suzhou 215011, China; 2 College of Pharmacy, Oregon State University, 2730 South Moody Avenue, Portland, OR 97201, USA

**Keywords:** ferrozine assay, iron quantification, colorimetric analysis, assay reproducibility, tube safety, digestion optimization

## Abstract

Accurate quantification of iron is essential in biological, chemical, and nanomaterial research, yet commonly used ferrozine-based assays suffer from safety hazards, inconsistent reduction efficiency, and unstable absorbance readings. To address these issues, we systematically optimized the classical protocol and validated improvements that enhance both operational safety and analytical reproducibility. In this work, samples were digested using perchloric acid and hydrogen peroxide, reduced with hydroxylamine, and complexed with ferrozine, with all steps quantitatively evaluated to identify conditions that minimize variability. The optimized assay introduces three key refinements: combining the two traditional hydroxylamine additions into a single reduction step, extending the post-complexation incubation to 2 h to ensure complete formation of the Fe^2+^–ferrozine complex, and performing digestion exclusively in 5 mL screw-cap polypropylene tubes to eliminate tube-bursting events frequently observed with flip-cap formats. Kinetic analysis confirmed that absorbance at 562 nm reaches a stable plateau after 2 h, and the resulting standard curve exhibited excellent linearity (R^2^ = 0.9999). These improvements significantly enhance precision, safety, and ease of implementation. The refined method is broadly applicable and enables reliable quantification of iron in tissues, cultured cells, aqueous solutions, and iron-containing nanomaterials.

## 1. Introduction

Iron is an essential trace element involved in oxygen transport, redox homeostasis, and enzymatic catalysis in virtually all living organisms. Quantitative determination of iron is therefore fundamental in disciplines ranging from biochemistry and cell biology to environmental and materials sciences. Among the numerous analytical techniques available—including atomic absorption spectrometry, inductively coupled plasma mass spectrometry (ICP-MS), and electron paramagnetic resonance—the colorimetric ferrozine assay remains one of the most widely adopted due to its simplicity, sensitivity, and low cost [1,2,3].

The ferrozine reagent (3-(2-pyridyl)-5,6-bis(4-sulfonic acid)-1,2,4-triazine disodium salt) was first introduced by Stookey in 1970 [1], who demonstrated its high selectivity for Fe^2+^ through the formation of a magenta Fe^2+^–ferrozine complex with an absorption maximum at 562 nm. Carter subsequently adapted this principle for biological samples [2]. Over the next three decades, various modifications were proposed to accommodate diverse sample matrices. Riemer et al. (2004) developed a standardized version for cultured cells, integrating acid digestion and hydroxylamine reduction into a compact workflow with improved sensitivity [3]. Later work further optimized specific components of the assay: Berker et al. (2010) evaluated ferrozine complexation chemistry within antioxidant capacity measurements, refining reaction conditions for enhanced analytical performance [4], while Jeitner (2014) introduced an optimized protocol for dissolved iron that improved sensitivity and reduced interference, directly addressing key reproducibility issues inherent to earlier formats [5].

Despite these advances, three persistent challenges remain. First, the use of two sequential hydroxylamine additions to reduce Fe^3+^ to Fe^2+^ is prone to operator variability and unnecessary delay. Second, short incubation times (typically 20–30 min) often lead to incomplete complexation, resulting in fluctuating absorbance values. Third, sample digestion in small 1.5 mL microcentrifuge tubes under acidic conditions can cause pressure buildup and tube rupture, posing serious safety risks (Jeff’s protocol, the conventional ferrozine procedure described in Ref. [6]. During routine laboratory work, we observed that these limitations collectively compromise assay precision, efficiency, and safety.

While previous optimized ferrozine-based protocols have primarily focused on improving analytical sensitivity or mitigating specific interferences, less emphasis has been placed on the systematic integration of operational safety, workflow robustness, and reproducibility across diverse sample matrices within a single standardized procedure. In particular, safety considerations during acid digestion, as well as the cumulative effects of multiple minor procedural steps on assay reproducibility, are often mentioned only implicitly or addressed in an ad hoc manner. Consequently, many existing protocols remain sensitive to operator handling and sample heterogeneity under routine laboratory conditions. To address these issues, we re-evaluated the ferrozine assay workflow and introduced three key improvements: (i) a single, optimized hydroxylamine addition; (ii) an extended 2 h incubation ensuring reaction equilibrium; and (iii) the use of larger, screw-cap polypropylene tubes to prevent pressure-related accidents. This paper describes the improved method in detail, compares its performance to established protocols, and provides a reliable, safer alternative for iron quantification across various biological and chemical samples.

## 2. Experimental Design

The improved protocol consists of three main stages:

Sample digestion and reduction: Releasing iron from biological or chemical matrices and reducing Fe^3+^ to Fe^2+^ in an acidic environment using hydroxylamine.

Complex formation: Reacting ferrous ions with ferrozine reagent to form the Fe^2+^–ferrozine complex.

Colorimetric measurement: Measuring absorbance at 562 nm against Fe^2+^ standards.

**Figure 1:**
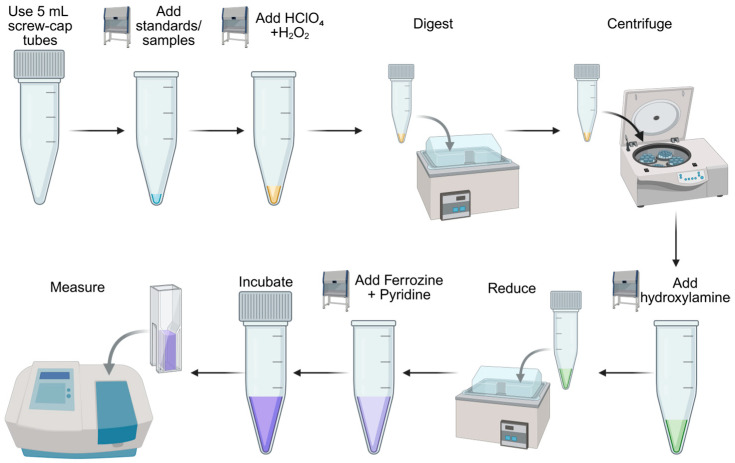
Workflow of the improved ferrozine-based iron quantification assay, showing digestion, reduction, complexation, incubation, and final spectrophotometric measurement. Created using Biorender.

The total duration from digestion to absorbance measurement is approximately 4 h, including incubation and cooling periods. Figure 1 provides a schematic summary of the workflow.

### 2.1. Materials

1. Ammonium iron(II) sulfate hexahydrate (Sigma-Aldrich, Merck KGaA, Darmstadt, Germany; Cat. No. 215406).
2. Perchloric acid (70%, Sigma-Aldrich, Merck KGaA, Darmstadt, Germany; Cat. No. 244252).
3. Hydrogen peroxide (30%, Sigma-Aldrich, Merck KGaA, Darmstadt, Germany; Cat. No. 95321).
4. Hydroxylamine hydrochloride (50%, Sigma-Aldrich, Merck KGaA, Darmstadt, Germany; Cat. No. 159417).
5. Ferrozine ((3-(2-pyridyl)-5,6-diphenyl-1,2,4-triazine-p,p′-disulfonic acid sodium salt)) (Sigma-Aldrich, Merck KGaA, Darmstadt, Germany; Cat. No. 160601).
6. Pyridine (Sigma-Aldrich, Merck KGaA, Darmstadt, Germany; Cat. No. 33553).
7. Deionized CO_2_-free water (prepared by boiling and cooling under nitrogen).
8. Screw-cap polypropylene tubes, 5 mL (Eppendorf, Hamburg, Germany).
9. Flip-cap polypropylene tubes, 5 mL (Eppendorf, Hamburg, Germany).
10. Microcentrifuge tubes, 1.5–2 mL (Eppendorf, Hamburg, Germany).
11. Screw-cap microtubes, 1.5–2 mL (Eppendorf, Hamburg, Germany).
12. Conical polypropylene tubes, 15 mL (Corning Inc., Corning, NY, USA).
13. Glass vials, 15 mL (VWR International, Radnor, PA, USA).

### 2.2. Equipment

- Water bath (Benchmark Scientific, Sayreville, NJ, USA; Cat. No. BSW3500).
- Tabletop centrifuge (Eppendorf 5702, Eppendorf, Hamburg, Germany; Cat. No. 5702000062).
- UV–Vis spectrophotometer (λ = 562 nm) (Shimadzu UV-1601, Shimadzu Corporation, Kyoto, Japan; Cat. No. 206-26000-92).
- Single-channel pipettes (10–100 μL, 100–1000 μL) (Eppendorf Research Plus, Eppendorf, Hamburg, Germany; Cat. Nos. 3123000041, 3123000068).
- Low-retention pipette tips (USA Scientific, Ocala, FL, USA; Cat. Nos. 1111-1830, 1111-2500).
- Chemical fume hood for handling acids and pyridine (Labconco Protector Echo, Labconco Corp., Kansas City, MO, USA).

## 3. Procedure

A schematic overview of the entire workflow is shown in Figure 1.

Major steps include

1. Sample digestion in HClO_4_ + H_2_O_2_ at 100 °C using 5 mL screw-cap polypropylene tubes.
2. Centrifugation to gather all reaction reagents.
3. One-step reduction of Fe^3+^ to Fe^2+^ with hydroxylamine at 37 °C.
4. Color development through complexation with ferrozine and stabilization by pyridine.
5. Two-hour incubation at room temperature to allow complete formation of the Fe^2+^–ferrozine complex.
6. Absorbance measurement at 562 nm and quantification using an Fe^2+^ standard curve.

This workflow can be applied to a wide range of sample types, including biological tissues, cultured cells, iron-containing solutions, and nanomaterials such as iron oxide nanoparticles or Fe-based metal–organic frameworks.

### 3.1. Tube Selection, Safety Evaluation, and Burst-Rate Testing

#### 3.1.1. Overview of Tube Types Evaluated

To identify a safe and reliable reaction vessel for high-temperature digestion, we systematically evaluated tubes that differed in

- Cap type: flip cap, flip cap equipped with a tube lock, and screw cap.
- Material: glass vs. polypropylene.
- Volume: 2 mL, 5 mL, and 15 mL.

**Figure 2:**
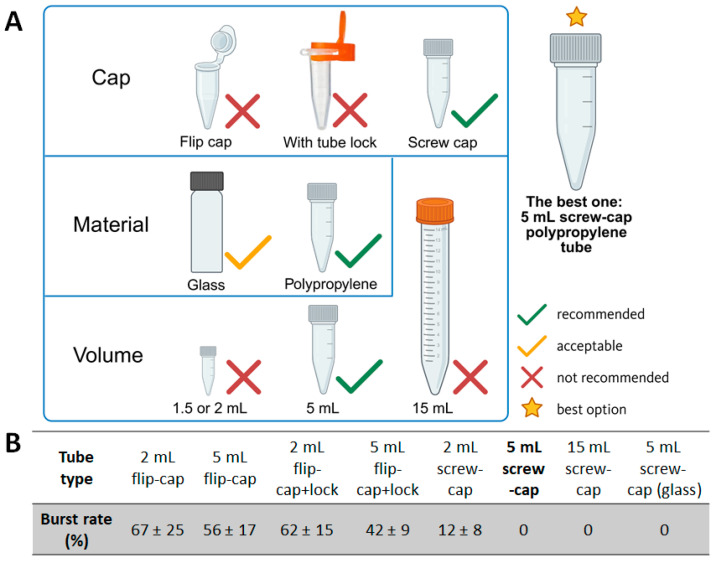
(**A**) Recommended tube types for safe and reproducible perchloric acid digestion. Created using Biorender. (**B**) Burst rates of different tubes.

The recommended and non-recommended tube configurations are summarized in Figure 2A.

#### 3.1.2. Experimental Determination of Burst Rates

All tube types were tested under the same digestion conditions:

A volume of 50 μL of 1 mM Fe standard solution + 50 μL 70% HClO_4_ + 50 μL 30% H_2_O_2_, heated at 100 °C for 30 min in a water bath. The number of tube ruptures or severe deformations was recorded to calculate the burst rate (mean ± SD; *n* ≥ 3 independent runs per tube type, 10 tubes in each test).

The burst rates are shown in Figure 2B and summarized below:

- Flip cap, 2 mL: 67 ± 25%.
- Flip cap, 5 mL: 56 ± 17%.
- Flip cap + tube lock, 2 mL: 62 ± 15%.
- Flip cap + tube lock, 5 mL: 42 ± 9%.
- Screw cap, 2 mL: 12 ± 8%.
- Screw cap, 5 mL: 0%.
- Screw cap, 15 mL: 0%.
- Screw-cap glass tube, 5 mL: 0%.

#### 3.1.3. Final Recommendation and Rationale

Collectively, the data indicate that cap design, material, and volume jointly determine tube safety during digestion:

- Both conventional flip-cap tubes and flip-cap tubes with a tube lock showed high burst rates (67 ± 25% and 62 ± 15%, respectively).
- Screw-cap tubes markedly reduced the incidence of rupture; no bursts were observed in either 5 mL or 15 mL screw-cap polypropylene tubes.
- Glass screw-cap tubes also prevented bursting but were ultimately deemed impractical because of their fragility, higher cost, and the need to transfer digested samples into polypropylene tubes before centrifugation—an extra step that increases complexity and potential handling error.
- Tube volume was also critical: although 2 mL screw-cap polypropylene tubes had much lower burst rates than flip-cap tubes, occasional failures still occurred. When the volume was increased to 5 mL or greater, the burst rate dropped to zero. However, 15 mL conical tubes, while safe, were operationally inconvenient and tended to deform in boiling water, sometimes making the cap difficult to reopen.

Taken together, these observations demonstrate that the 5 mL screw-cap polypropylene tube offers the best balance of safety, convenience, and cost. We therefore recommend this tube type as the standard reaction vessel for the optimized assay.

### 3.2. Preparation of CO_2_-Free Water

- Add an appropriate volume of deionized water to a glass bottle or Erlenmeyer flask.
- Boil the water vigorously for 15 min to remove dissolved CO_2_.
- Immediately cap the container while still hot and allow it to cool to room temperature.
- Use the CO_2_-free water within 24 h for preparing hydroxylamine and ferrozine solutions.

### 3.3. Preparation of Fe^2+^ Standards

- Prepare a 1.0 mM Fe^2+^ stock solution by dissolving ammonium iron(II) sulfate hexahydrate in CO_2_-free water. For applications requiring longer storage, the salt may be dissolved in CO_2_-free water containing 10 mM HCl to further suppress Fe^2+^ oxidation.
- Prepare working standards of 0, 0.05, 0.1 0.2, 0.4, 0.6, and 1.0 mM Fe^2+^ by diluting the stock solution with CO_2_-free water.
- Store standards on ice and use within the same day. Freshly prepared solutions are recommended for best reproducibility.

### 3.4. Sample Digestion

Note: Digestion is required for solid or complex matrices such as tissues, cells, and nanomaterials. For simple iron salt solutions, the digestion step can be omitted, and the assay can proceed directly to Section 3.5 (thus reducing the total reaction time from approximately 4 h to about 3 h).

- Label 5 mL screw-cap polypropylene tubes for all standards and samples.
- Pipette 50 μL of each standard or sample into the appropriate tube.
- Add 50 μL of 70% HClO_4_ followed by 50 μL of 30% H_2_O_2_ to each tube.
- Tightly close the screw cap. Ensuring a secure closure is critical to prevent cap opening during digestion.
- Place the tubes in a water bath preheated to 100 °C and digest for 30 min (Figure 1).
- Remove the tubes and cool them under running tap water or in a room-temperature water bath until they can be handled safely.
- Briefly centrifuge the tubes at 1000 rpm for 1 min to bring all liquid to the bottom of the tube.

### 3.5. Reduction of Fe^3+^ to Fe^2+^

- Prepare a 10% (*w*/*v*) hydroxylamine solution in CO_2_-free water.
- Add 150 μL of the hydroxylamine solution to each digested or undigested (solution-only) sample.
- Gently mix by flicking or brief vortexing.
- Incubate the tubes at 37 °C for 30 min in a water bath or heating block to ensure complete reduction.

### 3.6. Color Development

**Figure 3:**
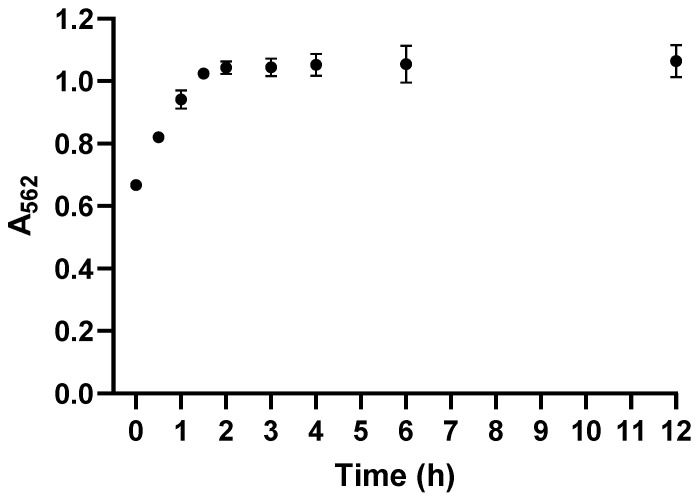
Time-dependent formation of the Fe^2+^–ferrozine complex from cell samples. Absorbance at 562 nm was recorded at the indicated time points after adding ferrozine to Fe^2+^ solution at RT. Data are presented as mean ± SD (*n* = 3).

- Prepare a 0.5% (*w*/*v*) ferrozine solution in CO_2_-free water.
- Add 500 μL of ferrozine solution (0.5%) to each tube.
- Add 500 μL of pyridine to each tube. Pyridine serves as a stabilizer to improve the stability of the Fe^2+^–ferrozine complex during incubation. Due to its toxicity, pyridine should be handled in a chemical fume hood with appropriate personal protective equipment.
- Mix gently to avoid bubble formation.
- Incubate the reaction mixtures at room temperature (RT) for 2 h. This incubation time was chosen based on kinetic analysis (Section 4.1; Figure 3), which demonstrated that Fe^2+^–ferrozine complex formation reaches a stable plateau within this period. While shorter incubation times may be sufficient for simple aqueous solutions, extended incubation ensures complete and reproducible complex formation for more complex biological matrices and nanomaterial-containing samples.

### 3.7. Measurement of Absorbance

**Figure 4:**
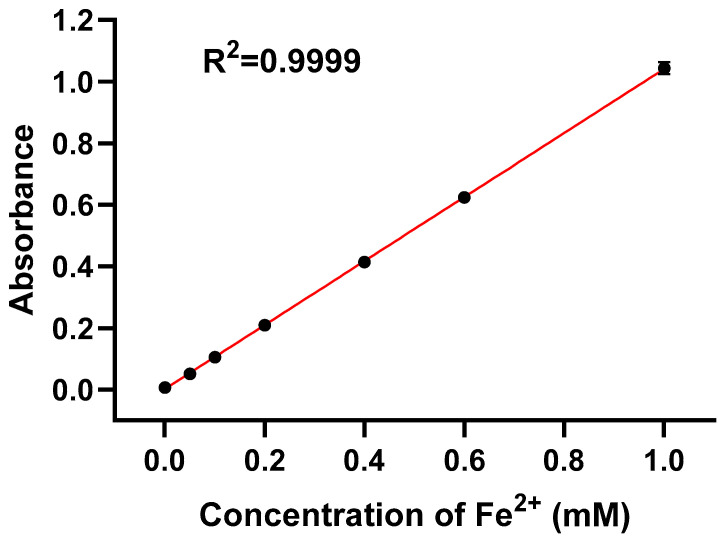
Standard curve for the ferrozine assay used to quantify iron concentration. The point at 0 mM represents the measured blank rather than a forced (0,0) point. Linear regression was performed without forcing the fit through the origin. the error bars correspond to the standard deviation (*n* = 3).

- If necessary, briefly centrifuge the tubes to collect any droplets from the tube walls.
- Transfer 1 mL of each sample to a cuvette or to the sample well compatible with the UV–Vis spectrophotometer.
- Measure absorbance at 562 nm against a reagent blank prepared in parallel without Fe^2+^.
- Construct a standard curve by plotting A_562_ versus Fe concentration for the Fe^2+^ standards (Section 4.2; Figure 4).
- Determine the iron concentration of unknown samples by interpolating their A_562_ values from the standard curve.
- For tissues, express iron content as mM or μg Fe per g wet tissue; for cell suspensions or nanomaterials, express as mM or μg Fe per sample or per mg of material as appropriate.

## 4. Expected Results

### 4.1. Kinetics of Fe^2+^–Ferrozine Complex Formation

The time-dependent formation of the Fe^2+^–ferrozine complex of cell samples is shown in Figure 3. Absorbance at 562 nm increased rapidly within the first 2 h, with the most pronounced changes observed during the first 90 min. Between 2 and 12 h, no further significant increase in absorbance was observed, indicating that complex formation had reached a plateau. Prolonging incubation from 30 min to 2 h significantly stabilized the absorbance signal. In the traditional method, OD_562_ drifted >20% between 30 and 90 min, whereas the new protocol maintained <2% variation across the same interval. This improvement reflects complete reaction equilibrium and reduced influence of residual oxidants.

Therefore, an incubation time of 2 h was selected for all subsequent measurements, as it ensures complete reaction and yields stable, highly reproducible absorbance values.

### 4.2. Standard Curve and Linear Range

The standard curve for the improved assay is shown in Figure 4. Within the 0–1.0 mM Fe^2+^ concentration range, the relationship between A_562_ and Fe^2+^ concentration is highly linear, with a correlation coefficient R^2^ = 0.9999, confirming excellent calibration performance.

Compared with the results we measured according to Jeff’s protocol [6], variability across triplicate measurements decreased from 15.2% ± 5.9% to below 3% (1.30–2.40% across the calibration range), reflecting a substantial improvement in reproducibility. These CV% values represent the intra-assay precision of absorbance measurements under stabilized reaction conditions and therefore quantify instrumental repeatability rather than sample-preparation variability.

To provide key analytical validation parameters, the limit of detection (LOD) and limit of quantification (LOQ) were determined according to the ICH Q2(R1) guidelines:

LOD = 3.3σ/S, LOQ = 10σ/S,

where σ = 0.00016 is the standard deviation of blank measurements and S = 1.038 is the slope of the calibration curve. The calculated LOD and LOQ were 0.51 μM and 1.54 μM, respectively, consistent with or superior to previously published values, as summarized in Table 1, which reported limits of detection for ferrozine-based iron assays vary widely depending on sample matrix, assay configuration, and degree of optimization. While highly optimized or matrix-specific systems can achieve submicromolar sensitivity, ferrozine assays applied to complex biological or food-related samples often exhibit substantially higher LODs due to matrix interference and background absorbance. The LOD achieved in this work (0.51 μM) falls within the central range of published values and represents a practical balance between analytical sensitivity, operational safety, and broad applicability.

Together, the submicromolar LOD, low-micromolar LOQ, and low CV% values indicate that the improved protocol provides enhanced sensitivity, precision, and reliability for iron quantification in diverse biological and chemical sample types.

### 4.3. Safety Performance

The optimized protocol markedly improves safety during perchloric acid and hydroxyl peroxide digestion. In the traditional method, heating 1.5–2 mL flip-cap microtubes with HClO_4_/H_2_O_2_ frequently leads to gas buildup, cap popping, acid leakage, or complete tube failure. In our evaluation, the Jeff protocol showed a failure rate of ~62–67%, posing clear chemical and physical hazards.

Using 5 mL screw-cap polypropylene tubes fully resolves this issue. These tubes provide a tighter seal, thicker walls, and greater headspace for pressure expansion. Across >300 digestions performed at 100 °C for 30 min, no rupturing, leakage, or cap failure occurred.

Overall, the improved tube system offers substantially enhanced safety, eliminates sample loss due to tube failure, and ensures consistent digestion quality for reliable iron quantification.

## 5. Applications

The improved ferrozine assay is broadly applicable and can be used to quantify iron in multiple types of samples:

- Biological tissues.

Homogenized tissues (e.g., liver, spleen, tumor) can be digested and analyzed. Iron content may be reported as mM or μg Fe per gram of wet tissue.

- Cultured cells.

Cell pellets from adherent or suspension cultures can be directly digested, reduced, and measured. Results can be normalized to cell number, protein content, or sample volume.

- Aqueous iron solutions.

The method is suitable for Fe^2+^ and Fe^3+^ solutions (the latter quantified after reduction) or for mixed-valence samples.

- Nanomaterials.

The assay is compatible with Fe-containing nanoparticles, such as iron oxide nanoparticles or iron-based metal–organic frameworks. After digestion, total iron content per mass of nanomaterial can be determined.

- Environmental and chemical samples.

Any water-soluble or digestible Fe source—including environmental water samples, catalytic reaction mixtures, or polymer–metal complexes—can be analyzed using this protocol.

## 6. Troubleshooting

Common problems and suggested solutions are summarized in the Table 2 as below.

## Figures and Tables

**Table 1:** Comparison of LOD values for ferrozine-based iron quantification methods.

No.	Reference	Sample Type	LOD (µM)	Key Notes
**1**	Nguyen & Waterhouse, 2019 [7]	Wine samples	0.36	Ferrozine/EDTA-based iron speciation in complex matrix
**2**	This work	Cells, tissues, nanomaterials	0.51	Tube-based ferrozine assay emphasizing safety and broad applicability
**3**	Chen et al., 2015 [8]	Aqueous standards	0.60–0.70	Ferrozine-based flow injection analysis (FIA)
**4**	Ferreira et al., 2021 [9]	Serum/food-related samples	0.61–0.64	Ferrozine photometric method with moderate sensitivity
**5**	Abramson et al., 2023 [10]	Aqueous (UCNP-assisted)	1.43–2.74	Ferrozine detection coupled with inner-filter effect
**6**	Akram et al., 2020 [11]	Food-related samples	79.3	Ferrozine assay with very high LOD due to matrix effects

**Table 2:** Common problems and suggested solutions.

Problem	Possible Cause	Recommended Solution
**Tube bursts during digestion**	Use of flip-cap tubes or other non-recommended formats	Use 5 mL screw-cap polypropylene tubes for all digestion steps.
**Low absorbance values**	Incomplete Fe^3+^→Fe^2+^ reduction or insufficient complexation time	Confirm hydroxylamine concentration and incubation at 37 °C for 30 min; ensure 2 h incubation after adding ferrozine and pyridine.
**High variability between replicates**	Multi-step hydroxylamine addition or inconsistent timing	Use the single-step hydroxylamine addition described here and strictly adhere to incubation times.
**Visible precipitate or turbidity**	Incomplete digestion of tissue/cell/nanomaterial samples	Extend digestion time, increase mixing, or gently vortex before centrifugation; verify that supernatant is clear before reduction.
**Non-linear standard curve**	Impurities or variable CO_2_ content in water; incorrect standard preparation	Prepare fresh CO_2_-free water; remake Fe^2+^ standards; ensure accurate pipetting.

## Data Availability

All data supporting the findings of this study are available within the article.
